# Evaluation of antivirals against tick-borne encephalitis virus in organotypic brain slices of rat cerebellum

**DOI:** 10.1371/journal.pone.0205294

**Published:** 2018-10-09

**Authors:** Nicole Lenz, Olivier Engler, Denis Grandgirard, Stephen L. Leib, Rahel Ackermann-Gäumann

**Affiliations:** 1 Institute for Infectious Diseases, University of Bern, Bern, Switzerland; 2 Biology Division, Spiez Laboratory, Swiss Federal Office for Civil Protection, Spiez, Switzerland; International Centre for Genetic Engineering and Biotechnology, ITALY

## Abstract

Neurotropic tick borne encephalitis virus (TBEV) causes life-threatening disease, and accounts for most cases of tick-transmitted viral infections in Central and Eastern Europe and Russia. No specific treatment for TBEV infections exists, and vaccination is recommended for people at risk. So far, various nucleoside analogues have been investigated in vitro as potential candidates for treatment of TBEV infections. However, in vitro experiments with more complex cell culture systems, such as organotypic culture slices which model the sophisticated architecture of the target tissue are lacking. Using TBEV as a model, we investigated the suitability of rat organotypic cerebellum slices (OCS) to study the effectiveness of nucleoside analogues with a well-known anti-TBEV activity. In these OCS, 50 μM of the nucleoside analogues 2’-C-methyladenosine (2’-CMA) and especially 7-deaza-2’-C-methyladenosine (7-deaza-2’-CMA) exhibited strong inhibitory effects on TBEV replication, reducing viral titers to an average of 10^3^-fold and TBEV RNA content 60-90-fold. In contrast, the influence of 2’-C-methylcytidine (2’-CMC) on TBEV replication was very weak, reducing virus titers by 10-fold and TBEV RNA content by 3-fold. In agreement with other studies, there was no noticeable difference in TBEV titers between OCS treated with 50 μM of Ribavirin and the DMSO treated controls. All tested nucleoside analogues exhibited excellent cytotoxicity profiles at concentrations of 50 μM. Our findings in OCS were highly comparable to data obtained in cell line culture systems. Therefore, OCS represent an ideal in vitro approach to study antivirals against TBEV and possibly other neurotropic viruses.

## Introduction

Tick-borne encephalitis virus (TBEV), a member of the *Flaviviridae* family, is a single stranded positive sense RNA virus with a genome of about 11 kb in length. It expresses a single polyprotein, which is post-translationally cleaved into 3 structural and 7 nonstructural proteins [1]. The principal vectors of TBEV are ticks, belonging to the *Ixodidae* family, more specifically *Ixodes ricinus* in Central Europe and *Ixodes persulcatus* in Eastern Europe and Russia [2]. Small rodents, deer, sheep and goats act as a reservoir for TBEV infection of ticks [3]. TBEV causes tick borne encephalitis (TBE); a disease caused by the European subtype virus is usually biphasic. It manifests in the first stage with febrile, flu-like symptoms, whereas in the second stage TBEV breaches the blood-brain barrier causing serious, potentially life threatening meningitis, encephalitis, meningoencephalitis, meningoencephalomyelitis and radiculitis. 10–20% of patients suffer from long lasting or permanent neuropsychiatric sequelae [4]. Although vaccinations against TBEV exist, TBE is still the most common tick-transmitted viral disease in Central and Eastern Europe as well as in Russia [3].

No specific antiviral treatment exists thus far, emphasizing the need for a safe and efficacious therapeutic intervention [5]. The mode of action of the largest class of antiviral drugs relies on inhibition of viral polymerases, for which nucleoside analogues are the most widely used [6]. Nucleoside analogues against flaviviruses act as inhibitors of RNA-dependent RNA polymerases (RdRp), methlytransferases, and helicases / nucleosid triphosphatases [7]. Eyer et al. carried out extensive studies investigating different nucleoside analogues and their influence on replication of TBEV strains Hypr and Neudoerfl in porcine kidney stable (PS) and human neuroblastoma cells (UKF-NB-4). 2’-C-methyl substituents to the nucleoside β-face resulted in the strongest inhibition of TBEV replication and had excellent cytotoxicity profiles. The most effective candidates were 2’-C-methyladenosine (2’-CMA), 2’-C-methylcytidine (2’-CMC) and 7-deaza-2’-C-methyladenosine (7-deaza-2’-CMA), all of which are inhibitors of the flaviviral RdRp [7–9]. The therapeutic effect of the most promising candidate, 7-deaza-2’-CMA, was furthermore tested in a lethal TBE mouse model. Mice treated with 7-deaza-2’-CMA presented with reduced viral titers in the brain and increased survival rates [10].

Cell lines enable screening of large libraries of antiviral compounds, yet the results vary considerably between different cell types [11]. Therefore we developed rat organotypic cerebellum slices (OCS), representing the primary target site for TBEV infection in the brain [12]. The natural anatomy of the cerebellum is conserved in OCS. Therefore they closely reproduce the complex interactions of different cell types which may play an important role in TBEV infection and treatment. To evaluate the OCS as an in vitro culture model for testing antiviral substances against neurotropic viruses, we evaluated the previously published inhibitory effect of the nucleoside analogues 2'-CMA, 2'-CMC, 7-deaza-2'-CMA and Ribavirin on TBEV replication in OCS. We could confirm the finding by Eyer et al. [8, 9] showing that, treatment with 2’-CMA and 7-deaza-2’-CMA significantly reduced TBEV titers, whereas 2' CMC resulted in only minimal reduction. Importantly, all investigated nucleoside analogues exhibited excellent cytotoxicity profiles in OCS.

## Materials and methods

### Ethics statement

Animal studies were approved by the Animal Care and Experimentation Committee of the Canton Bern, Switzerland (No. BE124/13 and BE142/16). Swiss National Guidelines for the performance of animal experiments were strictly followed.

### Porcine kidney stable (PS) cell cultures

PS cells, kindly provided by Daniel Růžek (Czech Academy of Sciences, České Budêjovice, Czech Republic), were cultured at 37 °C in Leibovitz (L-15) medium (Merck Millipore, Billerica, MA, USA) supplemented with 5% fetal calf serum, 1% penicillin-streptomycin and 1% glutamine (Sigma-Aldrich, St. Louis, MO, USA) [13].

### Production and maintenance of rat organotypic cerebellum slices (OCS)

Purkinje cells were identified as the main cell type infected by a TBEV model virus in the cerebellum [12]. Since Purkinje cells are most abundant and polarized in the brain of 10 days-old pups [14], we euthanized 10 days-old Wistar rats (Charles River Laboratories, Sulzfeld, Germany) by a lethal dose of intraperitoneal pentobarbital (G. Streuli & Cie. SA, Uznach, Switzerland). The brain was immediately removed and submerged in ice-cold dissection medium (Hanks balanced salt solution with 1% antibiotic-antimycotic, Gibco Life Technologies, Waltham, MA, USA, and 6mg/ml glucose, Sigma-Aldrich). The brains were cut in half in the sagittal plane and glued on a sample tray using Roti Coll Superglue (Carl Roth, Karlsruhe, Germany). To conserve the Purkinje cells’ dendritic trees, the cerebella were cut in 400 μm thin section in the sagittal plane by a Leica VT1000 S vibratome (Leica Biosystems, Wetzlar, Germany), while being submerged in ice-cold dissection medium. Four slices were transferred to one Transwell insert consisting of a semiporous (0.4 μm) membrane (Corning Inc., Corning, NY, USA). Inserts were placed in 6 well plates containing 1 ml serum-free Neurobasal medium (NBM) supplemented with 1% antibiotic-antimycotic, 1% Hepes, 1% Glutamax and 20 μl / ml B27 (Gibco Life Technologies). Slices were incubated at 37 °C and 5% CO_2_ for 9–10 days until further use. The medium was changed on the 1^st^ day and then every 2^nd^-3^rd^ day. For biosafety reasons, infected OCS were kept in a GenBox containing a CO_2_ bag to reach 5% CO_2_ (BioMérieux, Marcy-l’Étoile, France) without medium replacement until the end of the experiment.

### Virus strains and antiviral compounds

All experiments were performed with TBEV strain Hypr kindly provided by Daniel Růžek (Czech Academy of Sciences, České Budêjovice, Czech Republic). 2’-CMA and 7-deaza-2’-CMA were obtained from Santa Cruz Biotechnology (Dallas, TX, USA), while 2’-CMC and Ribavirin were from Sigma-Aldrich. The test compounds were solubilized in DMSO (sterile filtered, suitable for hybridoma, Sigma-Aldrich) to yield 10 mM concentrated stock solutions.

### Viral titer reduction assay

OCS were prepared as described above. Each test compound was assessed in 3 independent biological replicates (3 wells each containing 4 slices). One milliliter of supplemented neurobasal medium (NBM) containing 50 μl of each test compound at 50 μM (or 50 μl DMSO for the mock treated wells) and 10^5^ plaque forming units (PFU) of TBEV strain Hypr were used for infection. One hundred microliters of virus / antiviral solution was added on top of the OCS in droplets, whereas 900 μl of the solution was added to the bottom of the well. The plates were incubated for 1 h at 37 °C with a CO_2_ bag. The medium was replaced with 1 ml of supplemented NBM containing 50 μl of each test compound and incubated at 37 °C with a new CO_2_ bag for 72 hrs. Three OCS in 600 μl of supernatant were transferred to tubes containing ceramic beads with 1.4 mm diameter (CK14 tubes, Precellys, Bertin Technologies, Montigny-le-Bretonneux, France). The samples were homogenized at 6500 rpm twice for 25 seconds with a 10 second break. The tubes were centrifuged for 1 minute at 10’000 g and stored at -80 °C. One hundred microliters of homogenate was directly inactivated in 400 μl of AVL viral lysis buffer (Qiagen, Venlo, Netherlands) for RNA quantification. Remaining homogenate and supernatant were stored at -80 °C for plaque assay and cytotoxicity tests, respectively.

### Growth curves

Two different growth curves were performed. The first growth curved analyzed viral RNA content in supernatant (with the advantage of observing the same well at different time points) and the second assessed viral titer using a homogenization protocol optimized for virus particle recovery (rationale see results and discussion).

For the first growth curve we assessed titers using three independent biological replicates. Three wells containing four OCS were infected as described above. The medium was replaced with 1.05 ml of supplemented NBM. Fifty microliters of supernatant were directly inactivated in 200 μl of AVL viral lysis buffer (Qiagen). During the following four days 50 μl of supernatant were inactivated in AVL buffer (Qiagen) and replaced by 50 μl of supplemented NBM. RNA was extracted from the inactivated samples and eluted in half of the volume used throughout the study to compensate the reduced sample volume.

For the second growth curve, six wells of OCS were infected as described above. On days 0, 2, and 4, three OCS from one well were mixed with 600 μl of supernatant and homogenized as descried above, using 5000 rpm instead of 6500 rpm. To demonstrate reproducibility, on day 3 we performed this procedure using three independent biological replicates, i.e. three different wells of OCS. One hundred microliters of homogenate were directly inactivated in 400 μl of AVL buffer (Qiagen) for RNA quantification. The remaining homogenate was stored at -80 °C and later used for plaque assay.

### RNA extraction and qRT-PCR

RNA was extracted using the EZ1 Advanced robot and the EZ1 Virus Mini Kit v2.0 (Qiagen) according to manufacturer’s recommendations. qRT-PCR for detecting TBEV was based on a previously published protocol [15]. Primer and probe sequences amplifying a fragment of the envelope gene (TBEE-F6: GGCTTGTGAGGCAAAAAAGAA; TBEE-R2: TCCCGTGTGTGGTTCGACTT; TBEE-P4: FAM-AAGCCACAGGACATGTGTACGACGCC-BHQ-1) were ordered at Microsynth (Balgach, Switzerland). qRT-PCR was performed using TaqMan Fast Virus 1-Step Master Mix (Thermo Fisher Scientific, Waltham, MA, USA). The qRT-PCR conditions were as follows: 6.3 μl of 4x TaqMan Fast Virus 1-Step Master, 1 μl of each primer stock (10 μM), 0.6 μl of the probe stock (10 μM), 5 μl of sample, and RNase-free water to adjust the volume to 25 μl. PCR cycling included a reverse transcription step for 5 minutes at 50 °C, a polymerase activation step for 20 seconds at 95 °C, and 45 cycles of two-step cycling for 3 seconds at 95 °C and 30 seconds at 60 °C. Every sample was assessed in duplets and the average was calculated using the arithmetic mean. Genome equivalents/ml were calculated using the following formula: 10^(Ct value-slope)/intercept^ *100/5 (for 5 μl input) with the slope being -3.296 and the intercept 37.19 obtained from [16]. In order to assess the effect of premature chain termination on quantification of viral RNA copies, a second qRT-PCR system was used. This system called "TBEBRC" amplifies a fragment of the NS5 gene located near the 3'-end of the genome (positions 9136–9255 according to accession number U27495.1, as opposed to positions 1329–1416 for the fragment of the envelope gene). A detailed description of this second qRT-PCR system is given in the supplementary information file.

### Plaque assay

PS cell monolayers were used to assess viral titers based on a protocol of De Madrid et al. [17]. Twenty four well plates (TPP) were coated with poly-L-lysine according to manufacturer’s recommendations (Sigma Aldrich). 1.35 x 10^5^ PS cells in 300 μl of L15 medium (Leibowitz, Biochrom AG, Berlin, Germany) supplemented with 1% glutamine, 5% fetal calf serum, 1% penicillin-streptomycin and 0.5% neomycin were seeded into each well. Successively, 200 μl of supplemented L15 medium containing 10-fold dilutions of TBEV were prepared and used to infect the PS cells. Each dilution was assessed in duplets and the average was calculated using the arithmetic mean. After 4 h incubation at room temperature (RT), 400 μl of 1.5% carboxymethylcellulose (Sigma Aldrich) in supplemented L-15 medium was used to overlay the suspension. After 4 days of incubation at 37 °C, each well was washed with 0.7 ml of phosphate buffered saline (PBS) and stained with 0.7 ml of naphthalene black solution (1 g naphthalene blue black, 60 ml glacial acetic acid, 13.6 g sodium acetate in 1 L of H_2_O, Sigma Aldrich). The wells were stained between 35 and 90 minutes and washed once with 0.7 ml of H_2_O. Virus titer was assessed as PFU/ml.

### Immunofluorescence staining

One OCS per well was fixed in 4% formaldehyde in PBS for 90 minutes at RT and then stored in 18% sucrose in PBS at 4 °C. The OCS were put in tissue freezing medium (Leica Biosystems) and frozen at -20 °C inside a Jung CM 3050 S Cryostat (Leica Biosystems). The OCS were re-sectioned into 12 μm thick slices, mounted on adhesive Histobond glasslides (Marienfeld) and let to dry. Once dried (white appearance), the slices were submerged in PBS. Slides were washed three times with PBS, followed by staining with TrueBlack (Biotium, Fremont, CA, USA) to quench autofluorescence. Slides were again washed three times in PBS and incubated overnight at 4 °C with primary antibodies directed against flavivirus group specific antigens (2 μg/ml, mouse monoclonal, Millipore), Purkinje cells (PCP-2, 4 μg/ml, rabbit polyclonal, Santa Cruz Biotechnology) or mature neurons (FOX3, 1 μg/ml, rabbit polyclonal, Millipore). Tris buffered saline (pH 7.6) containing 0.25% bovine serum albumin was used as antibody diluent. Sections were washed three times with PBS and then stained with the secondary antibodies anti-rabbit-Alexa Fluor 488 (2 μg/ml, Life Technologies) and anti-mouse-Alexa Fluor 594 (1 μg/ml, Jackson ImmunoResearch, West Grove, PA, USA) for 60 minutes at RT in the dark. The OCS were washed three times with PBS and mounted with VectaShield containing Dapi (ReactoLab, Servion, Switzerland). Images were acquired on a Zeiss Observer Z1 using the ZEN 2.3 software and the MosaiX table for scanning large tissue sections (Zeiss, Oberkochen, Germany).

### Cytotoxicity test

To detect cytotoxicity of the compounds alone or in combination with TBEV infection, we measured lactate dehydrogenase (LDH) release into culture supernatants using the LDH Cytotoxicity Assay Kit (Pierce, Thermo Fisher). The manufacturer’s recommendations were strictly followed and each supernatant was assessed in triplicates. For cytotoxicity testing of the compounds without TBEV infection, OCS were treated with specified concentrations of nucleoside analogues following the same procedure as TBEV-infected OCS. Briefly, 100 μl of the nucleoside analogue in culture medium was added in droplets on top of the OCS and 900 μl was added below the insert. DMSO without the nucleoside analogues was used as control. This was incubated for 1 hr with a CO_2_ bag, followed by a complete medium change containing the nucleoside analogue and incubation for 72 hrs with a new CO_2_ bag.

Absorbance was measured with a GloMax Luminometer (Promega, Madison, WI, USA) at 490 nm and at 600 nm (background noise).

### Statistical analysis

A two-sided, unpaired student's t-test was applied to assess statistical significance comparing viral titer reduction and cytotoxicity during TBEV infection by nucleoside analogues using the Bonferroni correction (α = 0.0125, n = 3). Calculations were performed using Microsoft Office Excel 2016.

## Results and discussion

Four nucleoside analogues were investigated for their inhibitory effect on the replication of TBEV strain Hypr in rat OCS. OCS have the advantage of comprising the entire cell community with the conserved natural structure occurring in the cerebellum of suckling Wistar rats.

**Fig 1:**
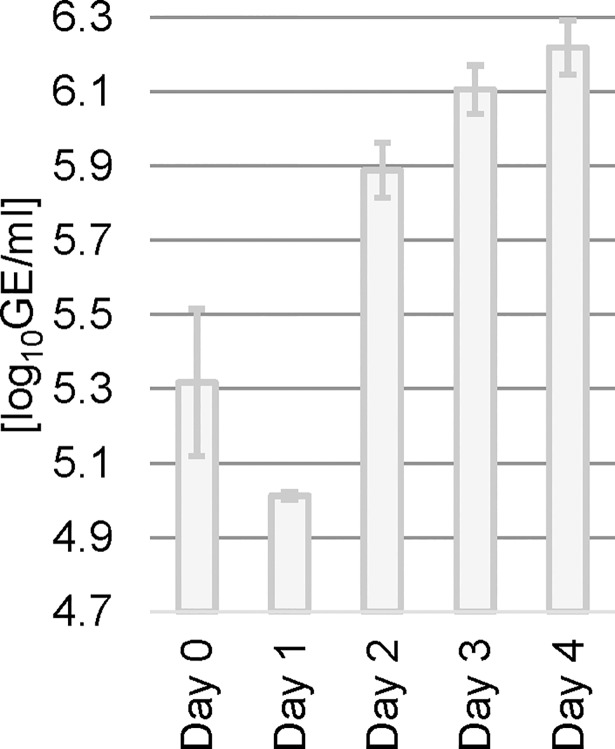
Growth curve of TBEV strain Hypr in OCS infected with 10^5^ PFU / ml and determined by qRT-PCR. Samples were taken from supernatant. Bars show arithmetic mean of three independent biological replicates and error bars indicate the standard deviation (n = 3). GE = genome equivalents.

Ribavirin, 2’-CMA, 2’-CMC and 7-deaza-2’-CMA were tested at a concentration of 50 μM equivalent to the study of Eyer et al. [8]. OCS were infected with 10^5^ PFU of TBEV strain Hypr. First, growth curves were established to determine the ideal harvesting time point (Fig 1). TBEV RNA content was assessed during four consecutive days in supernatant of three independent biological replicates. The sampling time points in Fig 1 do not exceed 4 days post infection, since a medium change necessary at latest on day 4 post infection would have implied an important impact on the culture system, including the removal of viral particles present in the medium. Based on these results, the samples for all subsequent experiments were harvested three days post infection. In a preliminary study, we observed that 3 days after infection with 10^5^ PFU/ml, TBEV titers are considerably higher in homogenized OCS than in supernatant (S1 Fig). Therefore, in the present study a mixture of OCS and supernatant (3 OCS in 600 μl of medium) was homogenized and then used for assessing TBEV replication. TBEV titer reduction was analyzed by qRT-PCR, plaque assay and immunofluorescence analysis.

### Inhibition of TBEV replication

**Fig 2:**
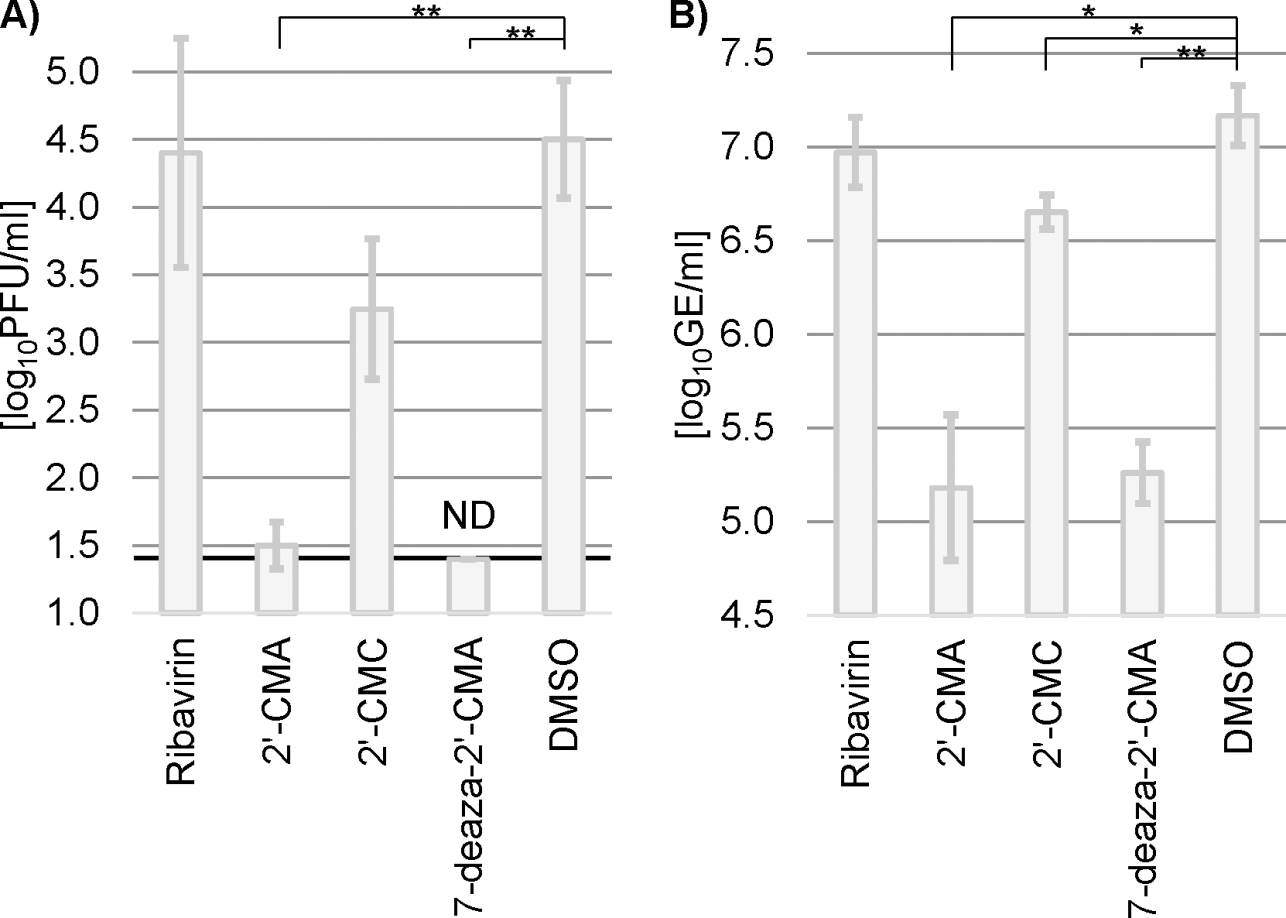
Reduction of TBEV titers by the specified nucleoside analogues measured in homogenate. OCS were infected with 10^5^ PFU of TBEV strain Hypr and treated with 50 **μ**M of each compound. TBEV titers were measured by plaque assay (A) and qRT-PCR (B) three days post infection. 50 **μ**l DMSO were added as mock treatment. The detection limit of the plaque assay was 1.4 log_10_ PFU / ml (thick black line). Bars show arithmetic mean of three independent biological replicates and error bars indicate the standard deviation (n = 3). PFU = plaque forming unit, GE = genome equivalents, 2'-CMA = 2'-C-methyladenosine, 2'-CMC = 2'-C-methylcytidine, 7-deaza-2'-CMA = 7-deaza-2'-C-methyladenosine, DMSO = dimethyl sulfoxide, ND = not detected, * = p-value < 0.0125, ** = p-value < 0.001.

Fig 2 shows the effect of the test compounds on TBEV titer as determined by plaque assay (Fig 2A) and RNA content as genome equivalents (GE) assessed by qRT-PCR (Fig 2B) in three independent biological replicates. The nucleoside analogues 2'-CMA and 7-deaza-2'-CMA presented with the highest potential to reduce TBEV titers and RNA content. qRT-PCR revealed a 75-80-fold decrease in TBEV GE compared to mock treated OCS (DMSO; p = 0.0012 for 2'-CMA and p = 0.00014 for 7-deaza-2'-CMA), while TBEV titers measured by plaque assay were reduced by approximately 10^3^-fold (p = 0.0004 for 2'-CMA and p = 0.0002 for 7-deaza-2'-CMA). In contrast, 2'-CMC exhibited only a weak anti-TBEV effect with a 3 fold decrease in GE (p = 0.0084) and TBEV titer decrease of about 10-fold (statistically not significant). Ribavirin had no significant effects on TBEV replication with results comparable to DMSO treated controls. Interestingly, the inhibitory effect of the nucleoside analogues was more pronounced in TBEV titers as compared to the measured RNA content. It is known, that the tested compounds inhibit the viral RdRp by sterically hindering the polymerase from incorporating more nucleosides, which results in premature chain termination and small TBEV RNA fragments starting from the 5'-terminus. The qRT-PCR we performed in Fig 2B used primers close to the 5'-terminus, thus theoretically able to detect the accumulation of prematurely terminated RNA fragments, which could be the reason for the distorted TBEV GE:PFU ratio. However, this effect was equally pronounced, regardless of whether a fragment near the 3'-terminus (1392–1416) or the 5'-terminus (9136–9255, according to accession number U39292.1) was amplified (S2 and S3 Figs and S1 Text). In addition to premature chain termination, 2'-CMA and 7-deaza-2'-CMA might have an additional mode of action. Such an effect has been documented for Ribavirin and hepatitis C virus on hepatoma cells. Ortega-Prieto et al. described, that Ribavirin reduced the specific infectivity of hepatitis C virus by induction of lethal mutagenesis, a process yielding a higher GE:PFU ratio, as observed in this manuscript [18].

**Fig 3:**
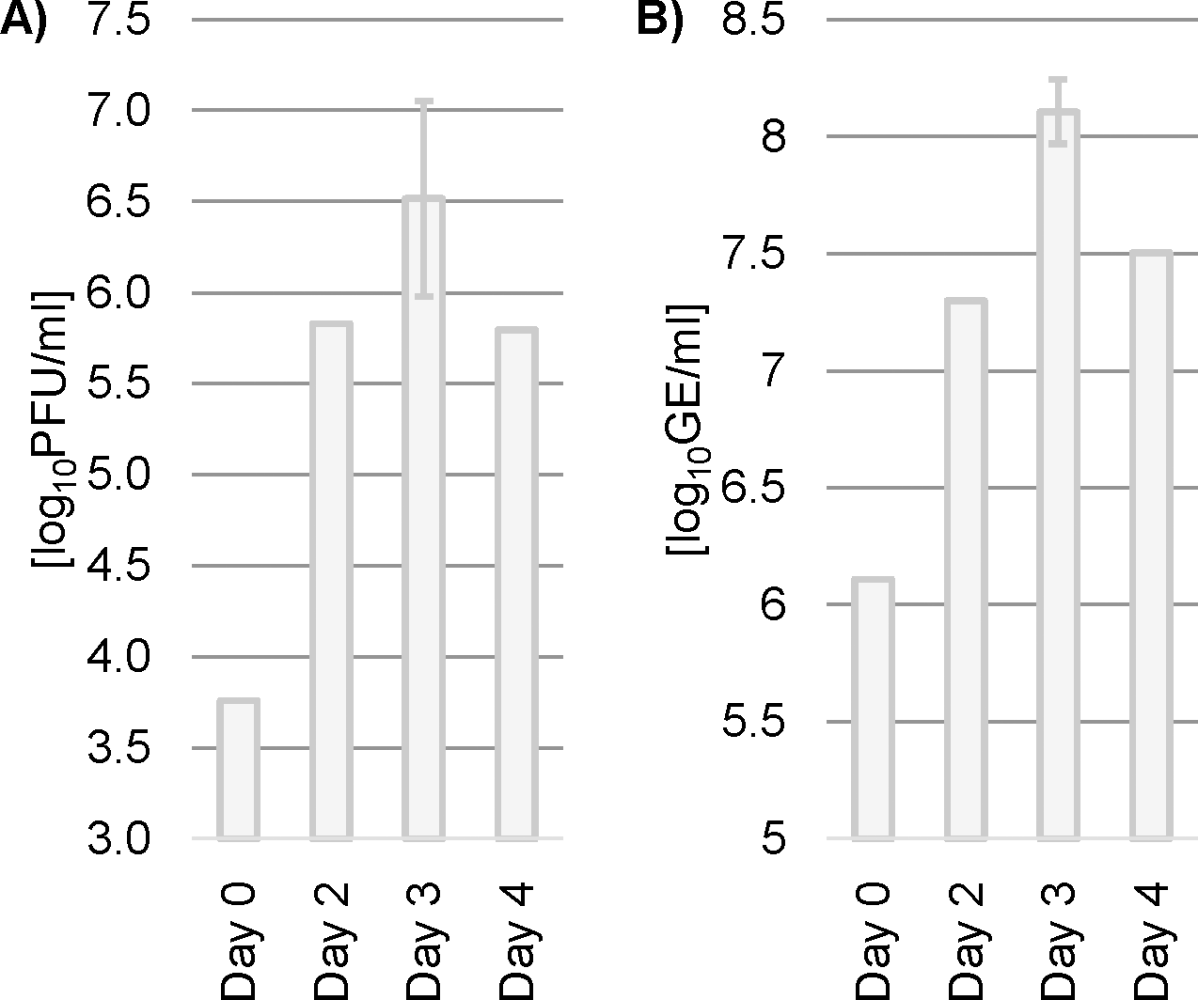
Repetition of the growth curve of TBEV strain Hypr applying an optimized homogenization procedure. Samples were taken from homogenate and TBEV titers were assessed by plaque assay (A) and qRT-PCR (B) three days post infection. Bars show either single data points or in case of day 3 the arithmetic mean of three independent biological replicates with error bars indicating the standard deviation. PFU = plaque forming unit, GE = genome equivalents.

Studies published by Eyer et al. based on the same nucleoside analogues, 2'-CMA, 2'-CMC and 7-deaza-2'-CMA, describe a TBEV titer reduction of 10^4^ to10^8^- fold in vitro when applied on PS and UKF-NB-4 cells and titer reduction of 10^5^-fold (7-deaza-2'-CMA), when applied in vivo to treat experimentally infected mice [8, 10]. The smaller reduction can be explained by the lower viral titer observed in OCS. Generally, the number of permissive cells in OCS is lower than in cell lines. Furthermore, a substantial amount of virus in the OCS was lost through the homogenization process. We observed, that homogenization at high speed resulted in accumulation of tissue lipids as a film on top of the aqueous solution. This lipid layer was excluded in further manipulations of the samples lowering the TBEV titers. Since the TBEV titer obtained by plaque assay of the 7-deaza-2'-CMA treated OCS was below detection limit, the detected inhibiting effect of the compound might be an underestimation. In an additional growth curve we measured optimized homogenate (using 5000 rpm instead of 6500 rpm) with qRT-PCR and plaque assay (Fig 3). For each time point we homogenized contents of one well and on day 3 we used three independent biological replicates (i.e. three different wells of OCS) to assess reproducibility. We retrieved on average 25 times more PFU and 7 times more GE. These results prove that a substantial amount of the viral RNA was associated and lost with the lipid layer in the homogenization process used for this study.

In comparison to Eyer et al. in PS cells, we found a less profound reduction of TBEV titers in OCS following treatment with 2'-CMC. Since a reduction of cell viability was observed in PS cells treated with 50 μM 2'-CMC, the amount in virus titer reduction could be due to cell toxicity related effects [8]. We confirmed the findings related to Ribavirin by Eyer et al. and revealed that at a concentration of 50 μM, Ribavirin exerted no influence on TBEV replication.

**Fig 4:**
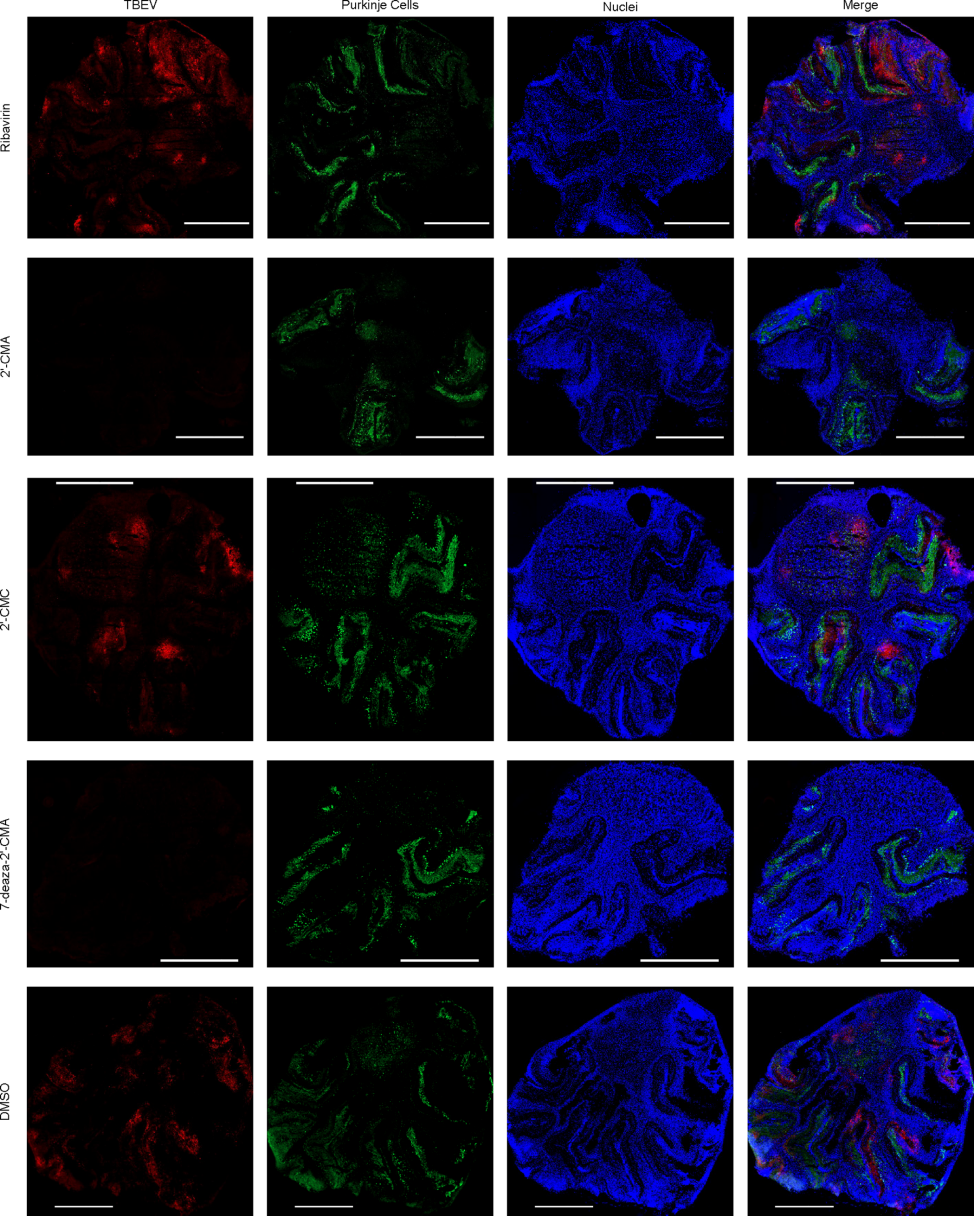
Immunofluorescence analysis of OCS infected with 10^5^ PFU / ml of TBEV strain Hypr and treated with the specified nucleoside analogue at 50 μM; 3 days post infection. Blue = Dapi staining, red = TBEV and green = Purkinje cells. 2'-CMA = 2'-C-methyladenosine, 2'-CMC = 2'-C-methylcytidine, 7-deaza-2'-CMA = 7-deaza-2'-C-methyladenosine, DMSO = dimethyl sulfoxide. Scale bars are 1 mm long.

To gain a visual impression we localized TBEV in OCS by immunofluorescence. OCS were stained for TBEV using a red fluorescent dye and for two abundant cell types present in the OCS, namely Purkinje cells and mature neurons in green (Fig 4 for Purkinje cells and S4 Fig for mature neurons). Similar to observations made with the TBEV model virus Langat [12], TBEV partially co-localized within Purkinje cells, the largest neurons present in the cerebellum (see S5 Fig for a close-up view). As expected, in OCS treated with 2'-CMA and 7-deaza-2'-CMA no TBEV could be detected, whereas in OCS treated with 2'-CMC and Ribavirin the fluorescent signal for TBEV was comparable to the controls. In summary, the immunofluorescence staining corroborates our previous findings and proves successful infection and replication of TBEV in OCS, as well as pronounced inhibition of viral infection and replication by 2'-CMA and 7-deaza-2'-CMA.

### Cytotoxicity of nucleoside analogues in OCS

**Fig 5:**
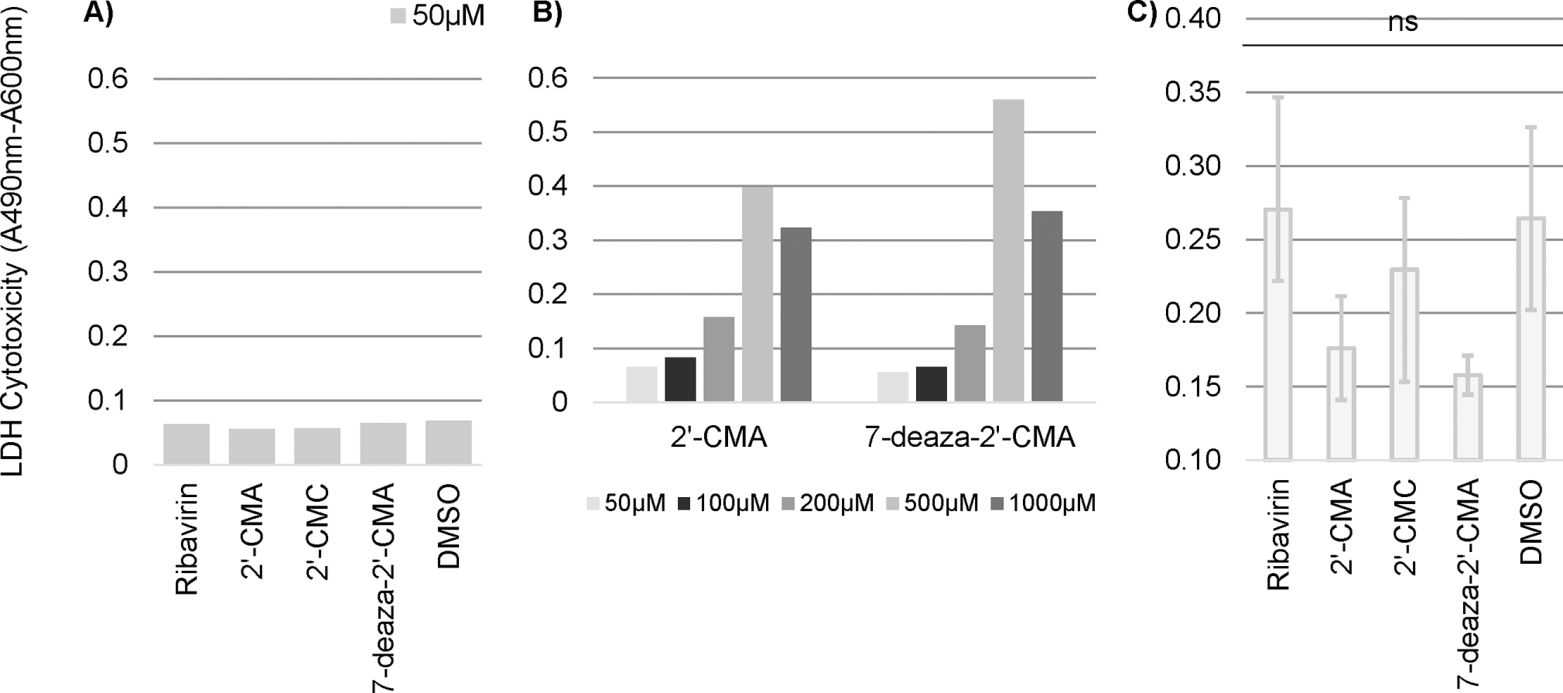
Cytotoxicity assessed by measuring LDH (Lactate dehydrogenase) release into supernatant of OCS 3 days after treatment with specified nucleoside analogues. (A) At 50 **μ**M, between 50 and 1000 **μ**M (B) and of TBEV infected OCS treated with nucleoside analogues at 50 **μ**M (C). Bars show either single data points (A and B) or the arithmetic mean of 3 independent biological replicates with error bars indicating the standard deviation (C). 2'-CMA = 2'-C-methyladenosine, 2'-CMC = 2'-C-methylcytidine, 7-deaza-2'-CMA = 7-deaza-2'-C-methyladenosine, DMSO = dimethyl sulfoxide, ns = not statistically significant.

The cytotoxicity profiles of Ribavirin, 2'-CMA, 2'-CMC and 7-deaza-2'-CMA were evaluated in uninfected (Fig 5A and 5B) and TBEV infected OCS (Fig 5C) by examining the culture supernatant for presence of LDH, a common marker for tissue damage. Cytotoxicity of each nucleoside analogue at a concentration of 50 μM on uninfected OCS was generally low and comparable to the DMSO control (Fig 5A). Since 2'-CMA and 7-deaza-2'-CMA exhibited the best antiviral activity against TBEV titers, cytotoxicity was also assessed for higher concentrations (Fig 5B). No difference was observed in OCS treated with 50 μM and 100 μM of 2'-CMA and 7-deaza-2'-CMA. LDH concentrations doubled in OCS treated with 200 μM for both compounds and reached a peak at 500 μM. The reduced signal measured at 1000 μM could be explained by an early release of most LDH within the first hour, which was lost upon the following medium change (Fig 5B). In infected OCS, LDH release closely correlated to observed TBEV titers and is therefore likely attributable to the cytopathic effect of the virus (Fig 5C). Taking together, these findings further substantiate, that, 2'-CMA and 7-deaza-2'-CMA exhibit a considerable inhibitory effect on TBEV replication in rat OCS at 50 μM and are likely safe at concentrations up to 100 μM. Compared to Eyer et al., we did not observe a cytotoxic effect of 2'-CMC higher than the DMSO control in uninfected OCS. This could explain the discrepancy observed in the effect of 2'-CMC on TBEV titers in OCS and PS and UKF-NB4 cell lines.

## Conclusions

Rat OCS represent an ideal in vitro approach to study antivirals' inhibitory effect on neurotropic TBEV replication in target tissue. 2'-CMA and especially 7-deaza-2'-CMA presented with excellent cytotoxicity profiles and substantial inhibitory effect on TBEV replication, thus identifying them as promising candidates for future evaluations of therapeutic interventions against TBEV infections.

Future steps encompass testing whether OCS are suitable to study antivirals against other neurotropic flaviviruses and assessing comparability to in vivo experiments.

## Supporting information

S1 Fig Comparison of TBEV titer in supernatant and homogenate. Titers were assessed by plaque assay. Bars show the arithmetic mean of two independent biological replicates with error bars indicating the standard deviation. Supe = supernatant, HG = homogenate.
(TIFF)

S2 Fig Comparison of TBEV RNA content using two different primer pairs. TBEE primer detect TBEV RNA close to the 5'-end, whereas TBEBRC measure RNA close to the 3'-end. Bars show the arithmetic mean of three independent biological replicates with error bars indicating the standard deviation. GE = genome equivalents.
(TIFF)

S3 Fig Ten-fold serial dilutions of untreated TBEV RNA from a PS cell culture measured by the two different primer pairs TBEE and TBEBRC. **Bars show the arithmetic mean of 2 dependent biological replicates.** GE = genome equivalents, ND = not detected.
(TIFF)

S4 Fig Immunofluorescence analysis of OCS infected with 10^5^ PFU / ml of TBEV strain Hypr and treated with the specified nucleoside analogue at 50 μM; 3 days post infection. Blue = Dapi staining, red = TBEV and green = mature neurons. 2'-CMA = 2'-C-methyladenosine, 2'-CMC = 2'-C-methylcytidine, 7-deaza-2'-CMA = 7-deaza-2'-C-methyladenosine, DMSO = dimethyl sulfoxide.
(TIFF)

S5 Fig Close-up view of immunofluorescence analysis of OCS infected with 10^5^ PFU / ml of TBEV strain Hypr to illustrate co-localization with Purkinje cells. Blue = Dapi staining, red = TBEV and green = Purkinje cells.
(TIFF)

S1 Text Description of additional experiments depicted in S2 and S3 Figs. (DOCX)
