# Supplementary figures and images for: Evaluation of antivirals against tick-borne encephalitis virus in organotypic brain slices of rat cerebellum
Source: PLoS One. 2018 Oct 9;13(10):e0205294. doi: 10.1371/journal.pone.0205294 (PMC6177190; doi:10.1371/journal.pone.0205294)

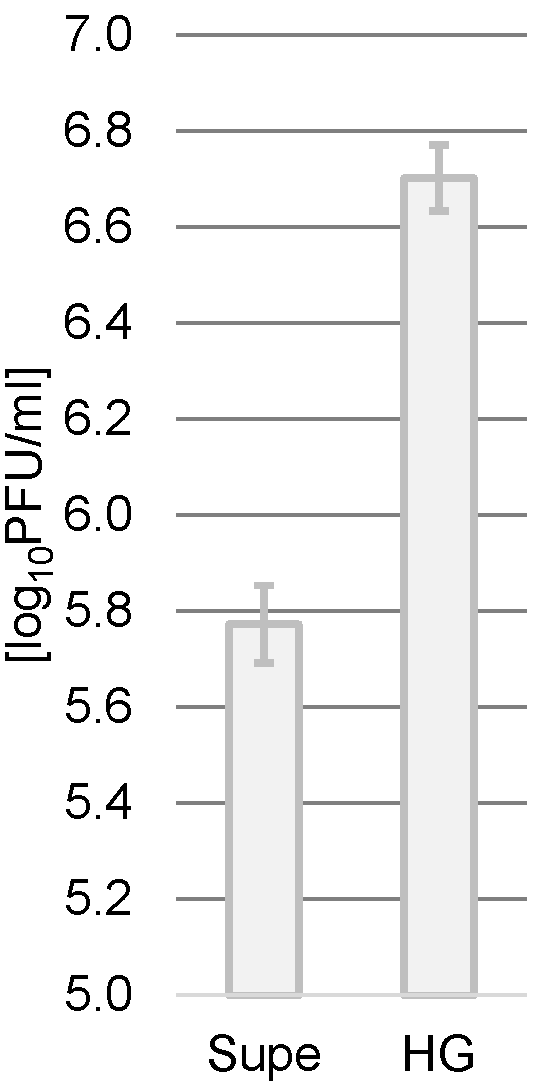

Supplement: S1 Fig — Titers were assessed by plaque assay. Bars show the arithmetic mean of two independent biological replicates with error bars indicating the standard deviation. Supe = supernatant, HG = homogenate. (TIFF) [file pone.0205294.s001.tiff]

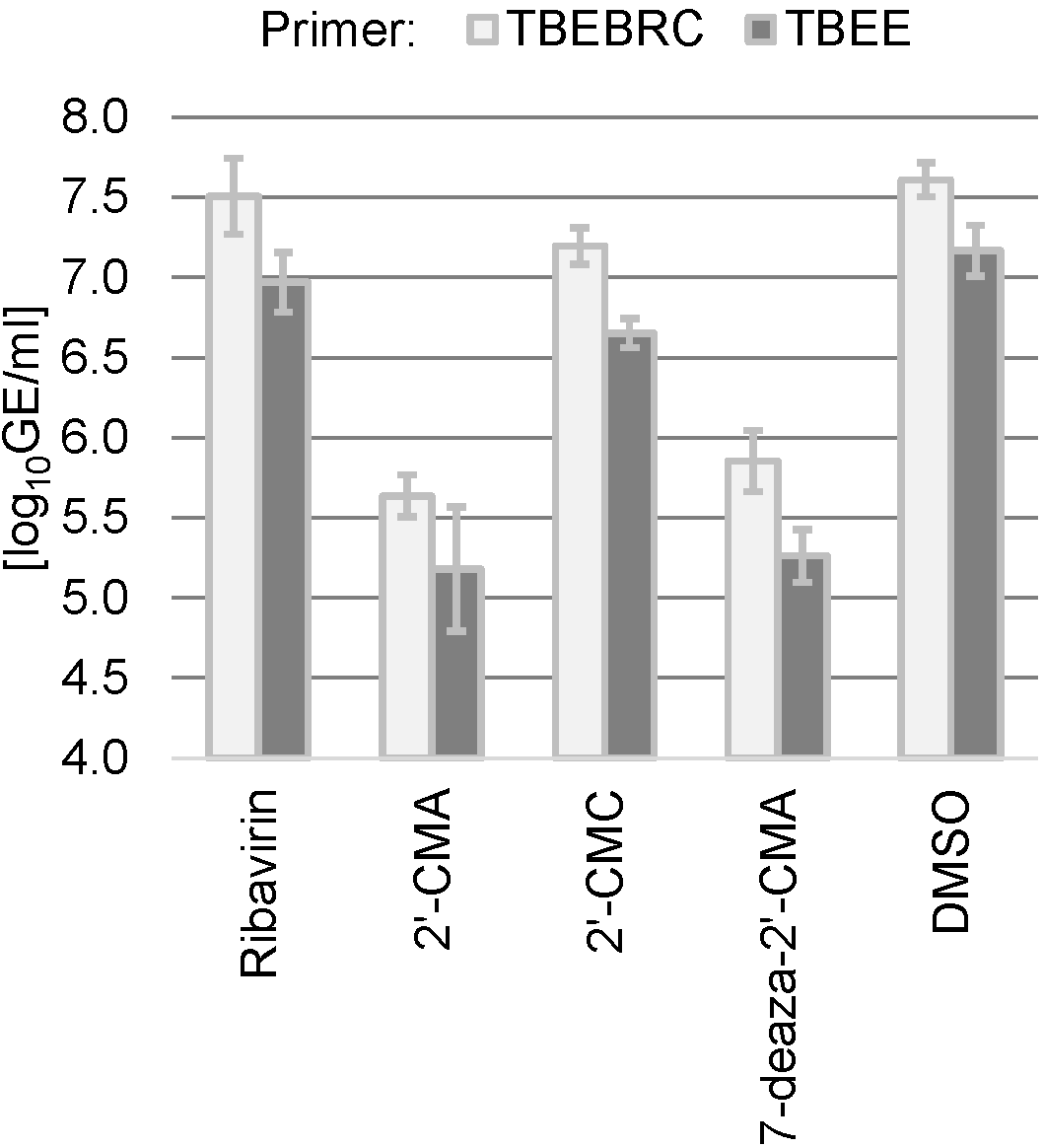

Supplement: S2 Fig — TBEE primer detect TBEV RNA close to the 5'-end, whereas TBEBRC measure RNA close to the 3'-end. Bars show the arithmetic mean of three independent biological replicates with error bars indicating the standard deviation. GE = genome equivalents. (TIFF) [file pone.0205294.s002.tiff]

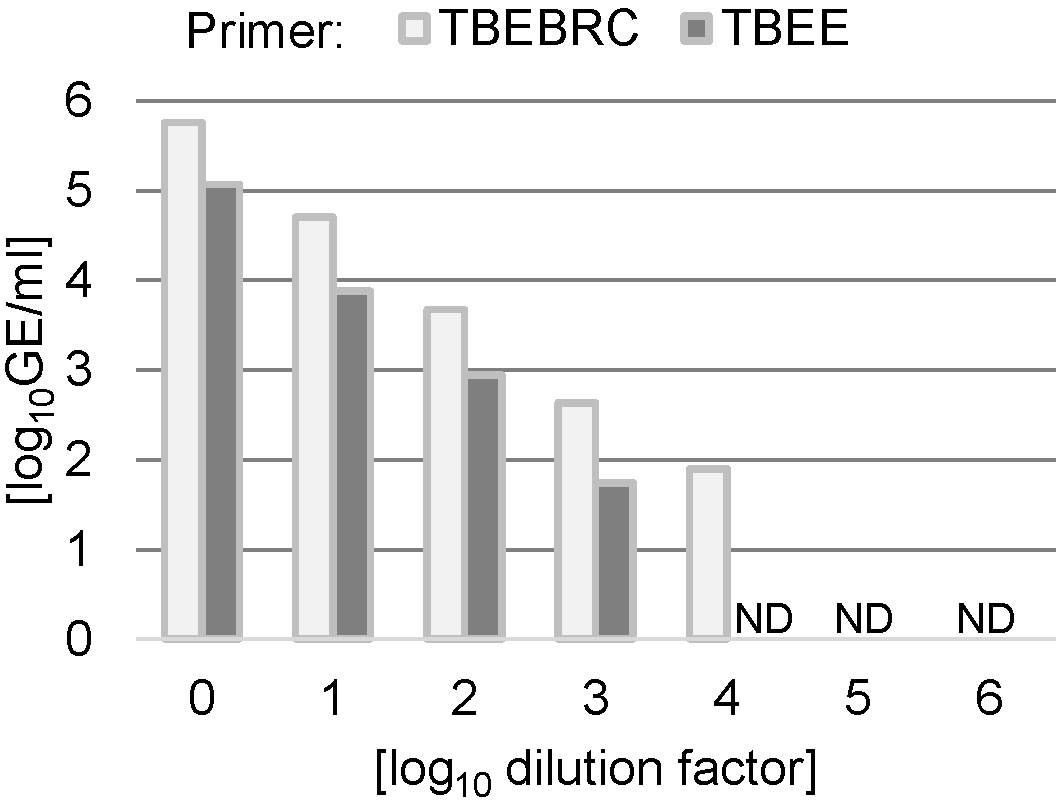

Supplement: S3 Fig — Bars show the arithmetic mean of 2 dependent biological replicates. GE = genome equivalents, ND = not detected. (TIFF) [file pone.0205294.s003.tiff]

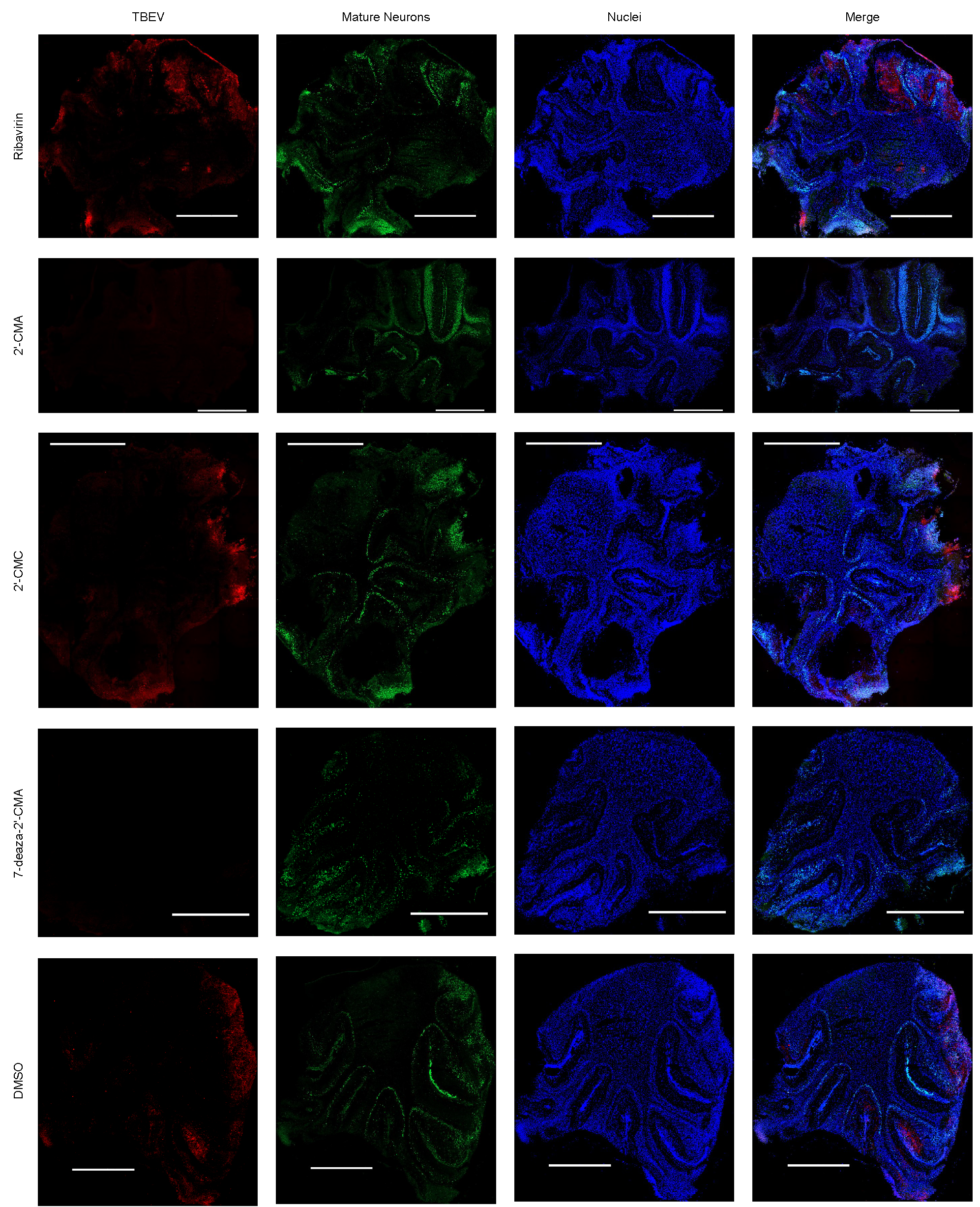

Supplement: S4 Fig — Blue = Dapi staining, red = TBEV and green = mature neurons. 2'-CMA = 2'-C-methyladenosine, 2'-CMC = 2'-C-methylcytidine, 7-deaza-2'-CMA = 7-deaza-2'-C-methyladenosine, DMSO = dimethyl sulfoxide. (TIFF) [file pone.0205294.s004.tiff]

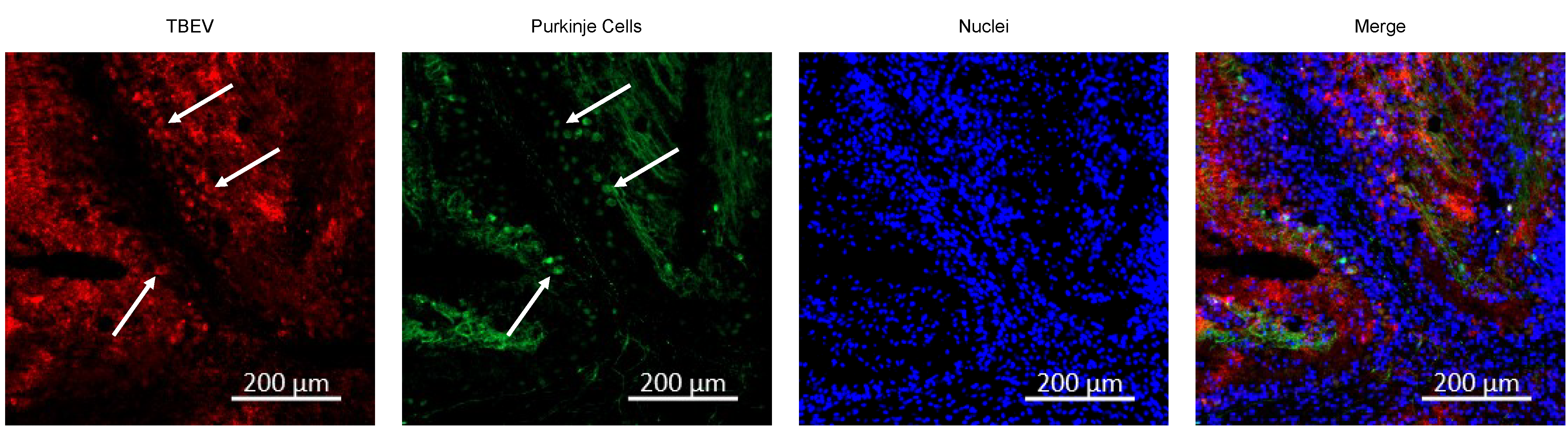

Supplement: S5 Fig — Blue = Dapi staining, red = TBEV and green = Purkinje cells. (TIFF) [file pone.0205294.s005.tiff]
