# Supplementary material for: Evaluation of antivirals against tick-borne encephalitis virus in organotypic brain slices of rat cerebellum
Source: PLoS One. 2018 Oct 9;13(10):e0205294. doi: 10.1371/journal.pone.0205294 (PMC6177190; doi:10.1371/journal.pone.0205294)
Supplement: S1 Text — (DOCX) [file pone.0205294.s006.docx]

**Supplementary Information**

**Supplementary methods**

qRT-PCR using primers TBEBRC was performed as described in the main text. Primer and probe sequences (TBEBRC-F2: GGATTCTTGAATGAAGACCATTGG; TBEBRC-R2: GAGTCCTCCATTCAGGGTTGAC; TBEBRC-P: FAM- TAAGCTTGAACTACCTGGGCTGGCACC -BHQ-1) were ordered at Microsynth (Balgach, Switzerland). Genome equivalents/ml (GE/ml) were calculated using the following formula: 10^(Ct value-slope)/intercept^ *100/5 (for 5µl input) with the slope being -3.5 and the intercept 41.443.

**Supplementary results and discussion**

We hypothesized, that the reason for the difference in inhibitory effect of the nucleoside analogues between plaque assay and qRT-PCR was due to the effect of premature chain termination during the RNA replication process. Since the fragment of the envelope gene amplified using the TBEE system is located near the 5'-terminus of the genome (positions 1392-1416 according to accession number U27495.1), it might have been possible that the production of this fragment was not as significantly influenced by the nucleoside analogs (random chain termination) as the production of the complete RNA genome (11'141 bases) and therewith the production of infectious particles. Therefore, we performed a second qRT-PCR with primers TBEBRC amplifying a fragment of the NS5 protein close to the 3'-end of the sequence (positions 9136-9255 according to accession number U27495.1) (S2 Fig). Contrary to our hypothesis, the quantity of viral RNA detected using this primer system was not lower than the one amplifying the fragment of the envelope gene. Most importantly, the differences in measured GE/ml using the two different qRT-PCR systems were constant among different treatments. Thus, treatment with 2'-CMA and 7-deaza-2'-CMA did not lead to accumulations of short viral 5' fragments detectable only by TBEE primers. We performed a dilution series with a TBEV Hypr RNA sample that was completely untreated, to prove that the increased viral RNA detected by TBEBRC primers was due to technical reasons (S3 Fig). Indeed, TBEBRC primers were more sensitive than TBEE primers and the calculated GE/ml detected using this system were 5-7 times higher than those detected using the TBEE system.
